# Loss of *Hairless* Confers Susceptibility to UVB-Induced Tumorigenesis via Disruption of NF-kappaB Signaling

**DOI:** 10.1371/journal.pone.0039691

**Published:** 2012-06-25

**Authors:** Hyunmi Kim, Alexandre Casta, Xiuwei Tang, Courtney T. Luke, Arianna L. Kim, David R. Bickers, Mohammad Athar, Angela M. Christiano

**Affiliations:** 1 Department of Genetics & Development, Columbia University, College of Physicians & Surgeons, New York, New York, United States of America; 2 Institute of Human Nutrition, Columbia University, College of Physicians & Surgeons, New York, New York, United States of America; 3 Department of Dermatology, Columbia University, College of Physicians & Surgeons, New York, New York, United States of America; Ohio State University Medical Center, United States of America

## Abstract

In order to model squamous cell carcinoma development *in vivo*, researchers have long preferred hairless mouse models such as SKH-1 mice that have traditionally been classified as ‘wild-type’ mice irrespective of the genetic factors underlying their hairless phenotype. The work presented here shows that mutations in the *Hairless* (*Hr*) gene not only result in the hairless phenotype of the SKH-1 and *Hr^−/−^* mouse lines but also cause aberrant activation of NFκB and its downstream effectors. We show that in the epidermis, *Hr* is an early UVB response gene that regulates NFκB activation and thereby controls cellular responses to irradiation. Therefore, when *Hr* expression is decreased in *Hr* mutant animals there is a corresponding increase in NFκB activity that is augmented by UVB irradiation. This constitutive activation of NFκB in the *Hr* mutant epidermis leads to the stimulation a large variety of downstream effectors including the cell cycle regulators cyclin D1 and cyclin E, the anti-apoptosis protein Bcl-2, and the pro-inflammatory protein Cox-2. Therefore, *Hr* loss results in a state of uncontrolled epidermal proliferation that promotes tumor development, and *Hr* mutant mice should no longer be considered merely hairless 'wild-type' mice. Instead, *Hr* is a crucial UVB response gene and its loss creates a permissive environment that potentiates increased tumorigenesis.

## Introduction

As a group, non-melanoma skin cancers (NMSC) are the most common type of cancer in the US with a combined incidence of over 2 million new cases annually [1]. Squamous cell carcinoma (SCC) is the second most common form of NMSC and is the most likely type to lethally metastasize, which makes understanding SCC development crucially important to human health [2]. In recent years, several genetic pathways have been implicated in the development of SCCs, particularly the ras and NFκB pathways ([3], [4], reviewed in [5]), but despite these advances, the molecular events leading to SCC development and progression remain poorly understood. Current research strategies utilize mice strains that lack hair to model carcinogenesis using either UV irradiation or chemical carcinogenesis since researchers prefer not to perform repeated shaving or depilation to visualize tumor development. Particularly favored strains feature mutations in the *Hairless* (*Hr*) gene (Entrez Gene ID 15460) that result in almost complete hair loss early in life [6].

*Hairless* mutant mice first appeared in the literature in the 1930's when they were described as having a striking wave of hair loss that occurs in a rostral to caudal manner starting at 3 weeks of age [6], [7], [8], [9]. Some fifty years later, *Hr* mutant mice were widely adopted as a convenient model for skin carcinogenesis studies and were discovered to be uniquely susceptible to both UV induced and chemical carcinogenesis [10], [11], [12]. Since then, the molecular basis of the Hairless phenotype has been established as either a proviral insertion into exon 6, as in the case of *Hr^hr^* and SKH-1 animals [13], [14], or point mutations, as is the case of the *Hr^rh^* animals [15], [16], [17]. Despite the knowledge that these strains have a mutated *Hr* gene, many carcinogenesis studies continue to use them and refer to unirradiated/untreated animals as 'wild-type hairless' mice.

Two early experiments using different *Hairless* mutant animals suggested that the increased susceptibility to carcinogenesis was, in fact, due to disruption of *Hr*. First, Iversen and Iversen (1976) showed increased tumorgenesis in *Hairless* mice bearing the *Hr/Hr* Oslo mutation as compared to wild-type, hairy littermates when both cohorts were exposed to a chemical carcinogenesis protocol. Secondly, Stoye *et al.* (1988) described the appearance and propagation of a revertant, hairy mouse (*Hr^hr+^*) that spontaneously lost the proviral insertion in the *Hr* allele. When used in both chemical and UV induced carcinogenesis protocols, the *Hr^hr+^* mice were found to have develop less tumors as compared to their *Hr^hr^* counterparts [11], [18]. Taken together, these genetic data suggested that *Hairless* is a key determinant of skin tumorigenesis.

Due to the overall phenotype of *Hairless* mutant mice, it has long been hypothesized that *Hairless* plays crucial roles in both the interfollicular epidermis and hair follicle. Supporting this hypothesis is the fact that *Hr* has been shown to be abundantly expressed in the suprabasal layers of the interfollicular epidermis, the matrix region of the hair follicle in catagen, and other follicular compartments such as the dermal papillae [19]. We (and others) have established a role for *Hairless* in the regressing catagen hair follicle [20], [21], [22], yet its function in the interfollicular epidermis has remained elusive. What has been deduced so far is that Hairless functions as a transcriptional co-factor that interacts with several nuclear hormone receptors such as the Vitamin D receptor (VDR) and thyroid hormone receptor (TR) (reviewed in [23]). In addition to a VDR/TR binding domain, Hr also contains a zinc finger domain, a nuclear localization signal and a putative Jumonji C domain (reviewed in [23]). Altogether, the presence of these multiple domains raises the possibility that Hr regulates multiple pathways thereby having pleotropic effects in the epidermis and influencing tumor susceptibility.

UV induced tumorigenesis occurs, in part, through the activation of the NFκB pathway and subsequent cellular stress and inflammation, therefore, one of the key UV response mechanisms is regulation of the NFκB pathway (reviewed in [24]). Furthermore, constitutive activation of the NFκB pathway is a hallmark of several types of tumors including SCCs [25], [26], [27] while its inhibition is a known chemoprevention for several tumor types (reviewed in [28]). Recent work from our lab has shown that in primary human keratinocytes *HR* is an early UVB response gene that regulates UVB induced NFκB activation thereby modulating cell cycling [29]. Due to these *in vitro* results and the clear role of the NFκB pathway in UV induced carcinogenesis, we postulated a connection between Hr and the NFκB pathway *in vivo*. Here, we present multiple lines of evidence demonstrating that disruption of the *Hr* gene leads to constitutive activation of the NFκB pathway, uncovering the molecular mechanism of the skin tumorigenesis susceptibility of *Hairless* mutant mice for the first time.

## Results

### *Hr* is rapidly upregulated in response to UVB and loss of *Hr* increases susceptibility to UVB induced tumorigenesis

**Figure 1:**
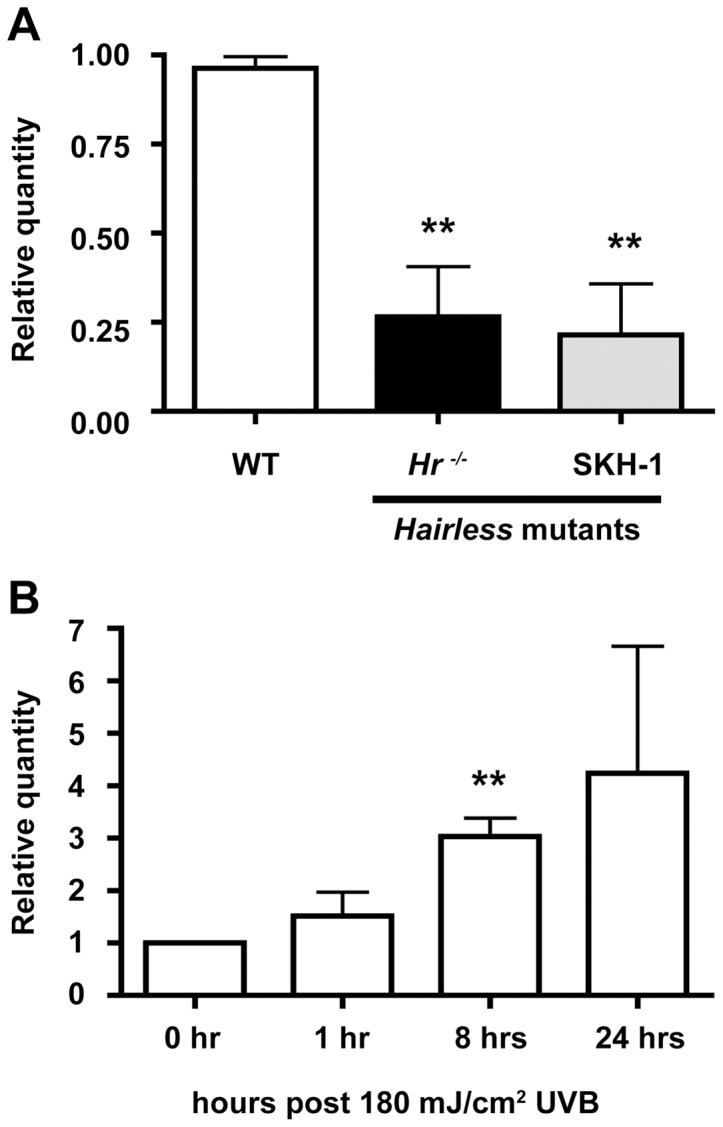
*Hairless* mutants have lower levels of *Hr*, an early a UVB response gene . A) *Hr* expression was measured in whole skin extracts by quantitative RT-PCR using primers designed to several locations in the *Hr* coding sequence. The *Hr^−/−^* and SKH-1 *Hairless* mutant strains have significantly decreased levels of *Hr* compared to WT. B) *Hr* expression was also measured in WT (*Hr^+/+^*) epidermis before and after UVB (100 mJ/cm^2^) exposure at 1, 8 and 24 hrs. *Hr* expression significantly increased at the 8 hr time point.

In order to place our studies in the context of earlier work, we first confirmed that the mouse lines SKH-1 and *Hr^hr^* (hereafter referred to as *Hr^−/−^*) both contained the mutation, a proviral insertion disrupting the open reading frame of *Hr* (Figure S1). We then quantified and compared the level of *Hr* mRNA from whole skin extracts of *Hr^−/−^*, SKH-1, and WT littermates. Both *Hr^−/−^* and SKH-1 animals exhibited significantly lower levels of *Hr* mRNA that equaled an ∼70% decrease in expression (Figure 1). Our previous work showed that *Hr* is expressed in suprabasal kerationcytes [19], [20] and is involved in the early UV response mechanism in primary human kerationcytes [29]. Therefore, we decided to focus our future experiments on the epidermis. In order to implicate Hr in the UV response pathway *in vivo*, we used real time PCR to evaluate *Hr* levels in the WT (*Hr ^+/+^*) epidermis at baseline as compared to 1, 8, and 24 hrs after a single, acute UVB dose. We observed a three-fold increase in epidermal *Hr* mRNA transcript levels from baseline (3.028±0.6147) as early as 8 hrs post UVB exposure (Figure 1B). Thus, *Hr* is rapidly transcriptionally activated in response to acute UVB suggesting that Hr plays a role in the immediate UVB response.

**Figure 2:**
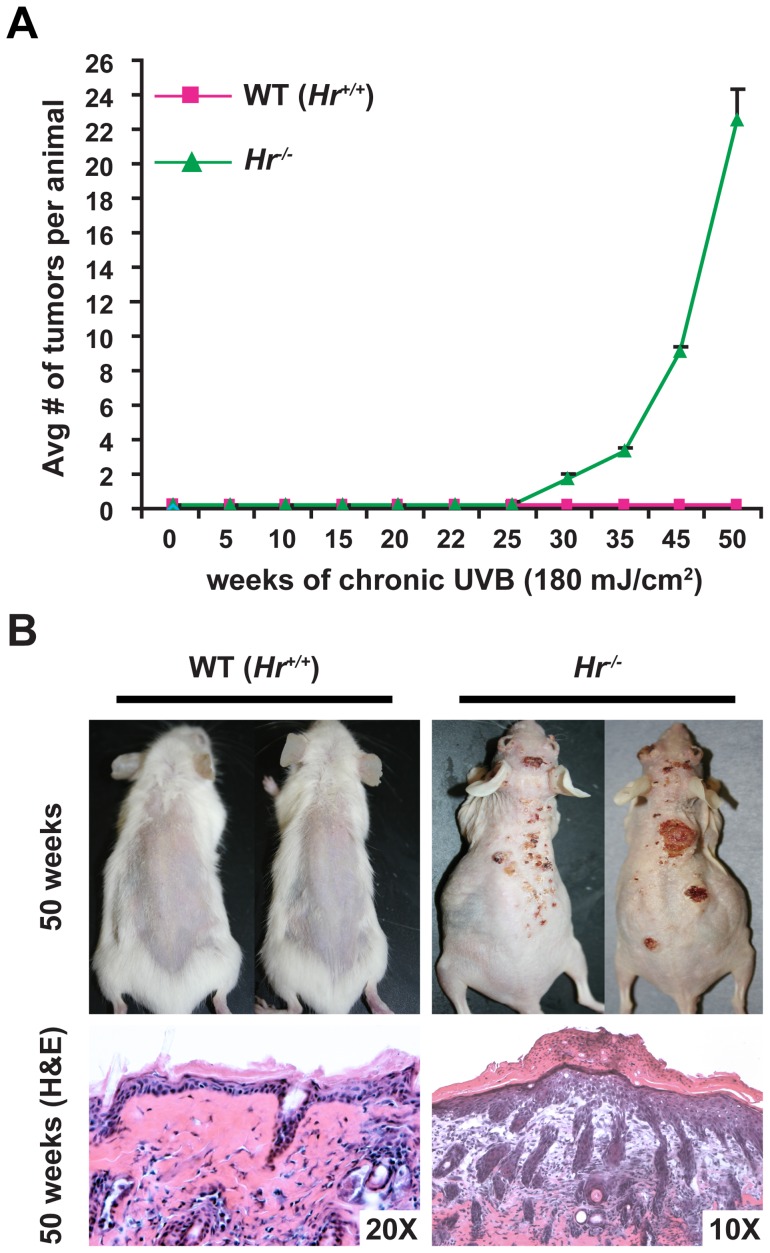
Mutations in *Hr* render animals susceptible to UVB-induced tumorigenesis. A) Female *Hr^−/−^* (n = 10) and age/sex matched WT littermates (*Hr ^+/+^*) were irradiated using 180 mJ/cm^2^ UVB three times a week for a total of 50 weeks and the number of tumors per animal were recorded on a weekly basis. WT littermates never developed tumors or papillomas, while *Hr^−/−^* animals developed tumors by 30 weeks. B) After 50 weeks of irradiation, *Hr^−/−^* mice had obvious tumors and histological analysis revealed that the majority of the tumors were aggressive SSCs. In contrast, WT animals did not develop any tumors even at the microscopic level.

To assess the impact of *Hr* mutations to UVB response, we performed a long-term UVB tumorigenesis protocol [180 mJ/cm^2^, the equivalent of 2 MEDS [30], [31], three times a week for a total of 50 weeks] using *Hr^−/−^* mutants and strain-matched WT (*Hr^+/+^*) animals. Neither WT (*Hr^+/+^*) nor *Hr^−/−^* animals showed any signs of papilloma formation at 22 weeks, however by week 30, *Hr^−/−^* animals began to develop small papillomas (Figure S2). 50 weeks into the protocol, the *Hr^−/−^* animals had an average of 22 tumors that necessitated the termination of the study (Figure 2). In contrast, WT (*Hr^+/+^*) animals showed no sign of tumor or papilloma formation at any time during the study.

Histological analysis of tissues obtained from the tumors in *Hr^−/−^* animals at 50 weeks indicated that they were highly aggressive SSCs, while there was no evidence of even microscopic tumor formation in WT (*Hr^+/+^*) skin (Figure 2B). Since the only known genetic difference between *Hr^−/−^* animals and WT (*Hr^+/+^*) littermates was the *Hairless* mutation (Figure 1), these results demonstrate that the disruption of *Hairless* was directly responsible for conferring tumor susceptibility.

### The *Hairless* mutant epidermis is hyperproliferative both basally and in response to UVB

Given the evidence in the literature and the results of our long-term UVB study, we next investigated the molecular role of *Hr* in the UVB response pathways. For these studies we used a single, acute UVB dose (180 mJ/cm^2^) and assessed early time points (1, 8, and 24 hrs post-irradiation) without the variable of secondary effects of a long term UVB protocol.

**Figure 3:**
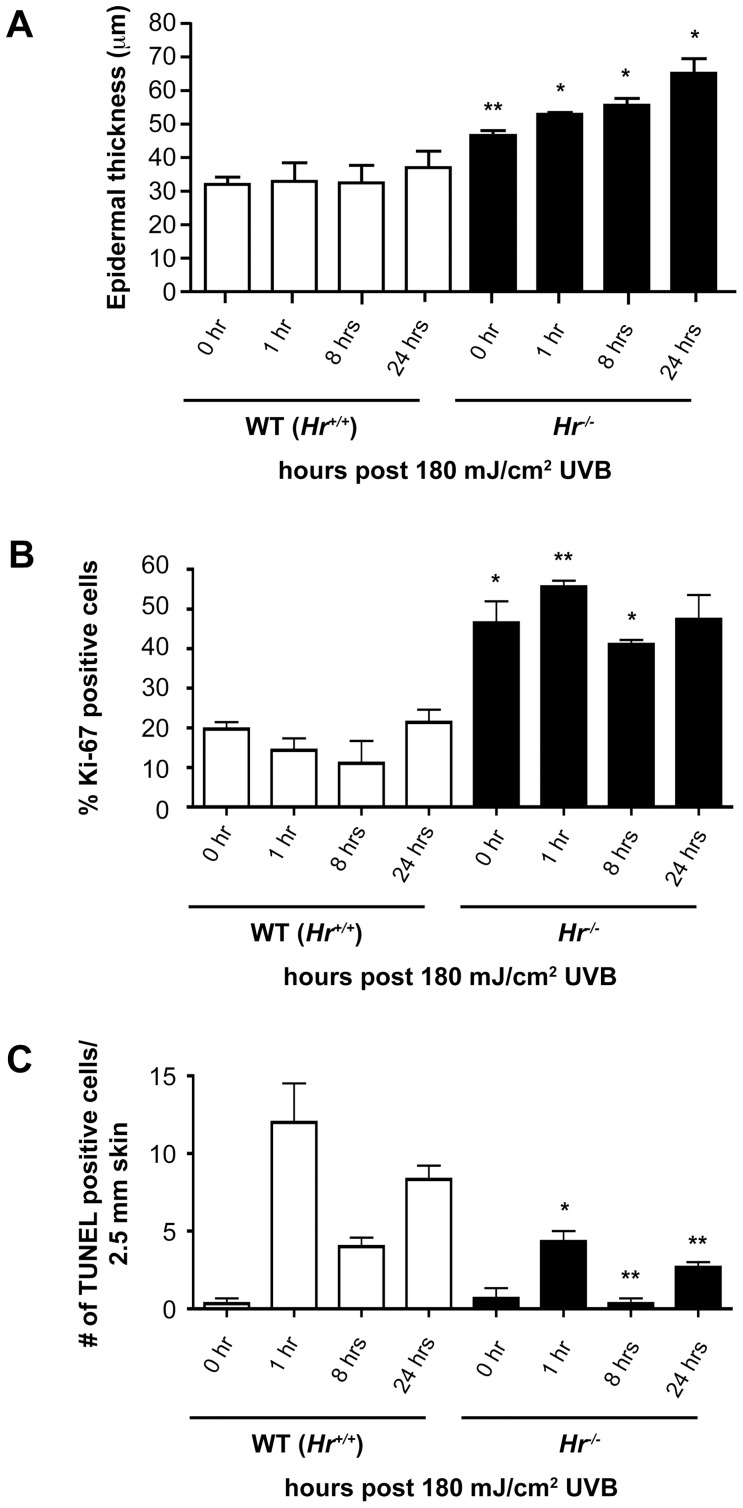
*Hairless* mutant epidermis is highly proliferative and exhibits a defective UVB response. A) Assessment of epidermal thickness using epidermal morphometry on H&E stained sections indicates that *Hr^−/−^* mice have significantly greater thickness prior to UVB (180 mJ/cm^2^) as compared to WT controls. Epidermal thickness remains elevated after UVB exposure. B) Skin sections were stained with Ki-67 to identify proliferating basal keratinocytes both at baseline and after UVB exposure at the specified time points. *Hr^−/−^* mice had a significantly higher percentage of Ki-67 positive cells at baseline as compared to WT and continued to have an elevated amount of Ki-67 positive cells after UVB. C) Skin sections were stained with TUNEL to identify apoptotic cells both at baseline and at the designated time points. The number of TUNEL positive cells was plotted per 2.5 mm section of skin. *Hr^−/−^* mice have a significantly lower number of TUNEL positive cells at 1 hr and 24 hr post UVB compared to WT.

Using epidermal morphometry, we found a significant increase in epidermal thickness both before and after UVB in *Hr^−/−^* mice as compared to wildtype conterparts (Figure 3A). At baseline, *Hr^−/−^* mice have a significantly thicker epidermis indicating the possibility of a hyperproliferative phenotype. Increased epidermal thickness in *Hr^−/−^* skin is maintained after UVB exposure with gradual increases at the indicated timepoints. We hypothesized that such an increase in epidermal thickness was due to either increased numbers of proliferating keratinocytes or a defect in apoptosis.

Therefore, we investigated the amount of proliferation found in the *Hairless* mutant epidermis by staining skin sections from *Hr^−/−^* and their respective WT littermates with the proliferation marker Ki67. As seen in Figure 3B, the percent of Ki67 positive cells found in the non-irradiated *Hr^−/−^* epidermis was significantly higher than in the WT epidermis [0.1969±0.0246 (WT) vs 0.4649±0.0772 (*Hr^−/−^*) as measured by the amount of Ki67 positive basal cells as a percentage of total basal cells). Throughout the time points sampled, the *Hr^−/−^* epidermis continued to exhibit a higher amount of proliferation than WT epidermis. Altogether this data indicated that the epidermal thickening seen in Figure 3A may, in part, be due to increased proliferation in the *Hairless* mutant epidermis.

In order to determine if the epidermal thickening could be partially attributed to defects in apoptosis, TUNEL staining was also performed (Figure 3C). We found that at homeostasis both WT and *Hr^−/−^* epidermis contained a low level of apoptosis that increased 1 hr after UVB exposure. However, the amount of apoptosis found in the *Hr^−/−^* epidermis was approximately 3 times less than in WT controls. This trend continued at 8 and 24 hrs with the level of apoptosis in the *Hr^−/−^* epidermis always being significantly less than that found in WT epidermis. When taken together with the proliferation data, this suggests that apoptosis may be impaired by loss of *Hr*; perhaps due to signals favoring proliferation or possibly because of the increased epidermal thickness partially shielding the keratinocytes from UVB irradiation.

### Loss of *Hr* leads to constitutively activated NFκB

**Figure 4:**
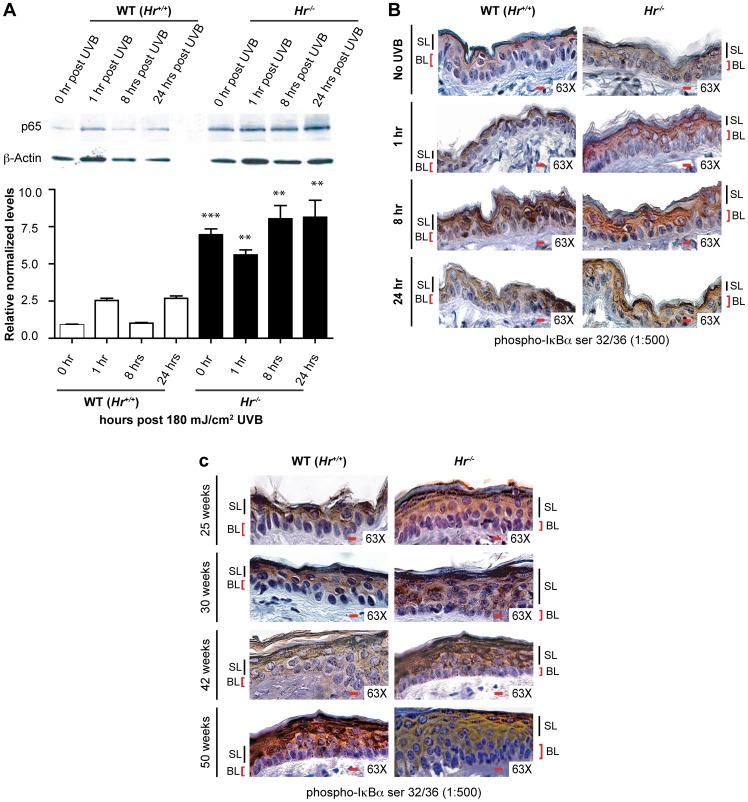
*Hairless* mutant epidermis shows constitutive activation of the NFκB signaling pathway. A) Western Blot and ImageJ densitometry analysis using anti-p65 antibody on isolated *Hr^−/−^* epidermal extracts after 1, 8 and 24 hrs post UVB (180 mJ/cm^2^) irradiation. B) Immunohistochemistry using p-IκBα antibody to determine the extent of NFκB signaling activity in the epidermis of WT (*Hr^+/+^*) and *Hr^−/−^* animals before and after a single, acute UVB (180 mJ/cm^2^) irradiation. IκBα is increased constitutively and after irradiation throughout the epidermal layer. C) Immunohistochemistry using p-IκBα antibody to determine the extent of NFκB signaling activity in the epidermis of WT (*Hr^+/+^*) and *Hr^−/−^* animals at the indicated time points during chronic UVB irradiation (180 mJ/cm^2^ UVB three times a week for a total of 50 weeks). IκBα is increased constitutively and after irradiation throughout the epidermal layer.

NFκB has long been known to play a role in UVB induced tumorigenesis and forms a nexus of proliferative and apoptotic pathways; additionally, our previous work shows that HR regulates NFκB *in vitro* [29]. We therefore theorized that Hr and NFκB may be mechanistically related in a similar fashion *in vivo*. To investigate a possible connection between the two proteins, we examined p65 protein levels in *Hr^−/−^* and matching WT (*Hr^+/+^*) littermate epidermis with and without acute UVB irradiation using Western blotting (Figure 4A). Prior to irradiation, *Hr^−/−^* epidermis had a significantly higher amount of the p65 subunit of NFκB. *Hr^−/−^* epidermis continued to express higher levels of p65 at 1 hr, 8 hrs, and 24 hrs post-UVB irradiation. Altogether this indicates that the lack of *Hr* in the *Hr^−/−^* epidermis results in a constitutively upregulated NFκB pathway.

In order to determine if the increase in p65 protein levels correlated with activation of the NFκB signaling pathway following acute UVB exposure, we examined *Hr^−/−^* and WT (*Hr^+/+^*) skin sections using an antibody against the phosphorylated inhibitor of NFκB signaling, IκBα. Activation of the NFκB complex is dependent on IκBα phosphorylation (p-IκBα) and subsequent degradation that allows nuclear translocation of the NFκB complex [32]. In addition to being upstream of NFκB, IκBα is also a known target gene that is transcriptionally regulated by NFκB [32]. Therefore, NFκB and IκBα form a positive feedback loop and p-IκBα, when assessed in conjunction with the overall increased levels of p65, can be used as an indicator to specifically localize the activated NFκB pathway [33].

Staining of non-irradiated *Hr^−/−^* skin showed the presence of p-IκBα throughout all the epidermal layers, including the basal layer, while in WT (*Hr^+/+^*) skin IκBα expression was restricted to the differentiated, suprabasal layers (Figure 4B). This clear demarcation of p-IκBα expression suggests that perturbation of NFκB signaling in basal keratinocytes may be responsible for the dysregulated proliferation and apoptosis observed in *Hairless* mutants. In response to acute UVB, we observed a gradual increase in the levels of p-IκBα in WT (*Hr^+/+^*) skin, with signaling in the basal keratinocytes beginning at 8 hrs post-UVB irradiation (Figure 4B). This expression pattern coincides with the time point previously observed to have a significantly higher amount of *Hr* mRNA in the WT epidermis after acute irradiation (Figure 1B). As expected, the intensity of p-IκBα signaling increased in response to UVB exposure in both the WT (*Hr^+/+^*) and *Hr^−/−^* mutant skin and by 24 hrs post UVB irradiation, both genotypes showed uniform p-IκBα staining in all layers of the epidermis with comparable intensity.

To study the role of NFκB activation in the increased susceptibility of *Hairless* mutant mice to tumorigenesis, we returned to the non-tumor bearing skin samples generated in the long-term irradiation study described in Figure 2. Interestingly, p-IκBα staining appeared in the basal and suprabasal layer of the mutant epidermis by week 25, a time point prior to tumor development (Figure 4C). In contrast, at the same timepoint, the WT (*Hr^+/+^*) epidermis continued to show only suprabasal localization of p-IκBα. By week 30 (when tumors were first observed on *Hr^−/−^* mice) the staining pattern remained the same in both sets of animals, though the *Hr^−/−^* epidermis had a higher intensity of staining. The intensity of the p-IκBα staining in the *Hr^−/−^* epidermis continued to increase at week 42 and plateaued at week 50, while the WT (*Hr^+/+^*) epidermis only began to show staining in the basal layer of the epidermis at week 42 which increased at week 50.

### Pharmacological inhibition of NFκB signaling reduces UVB induced tumorigenesis in *Hairless* mutant mice

**Figure 5:**
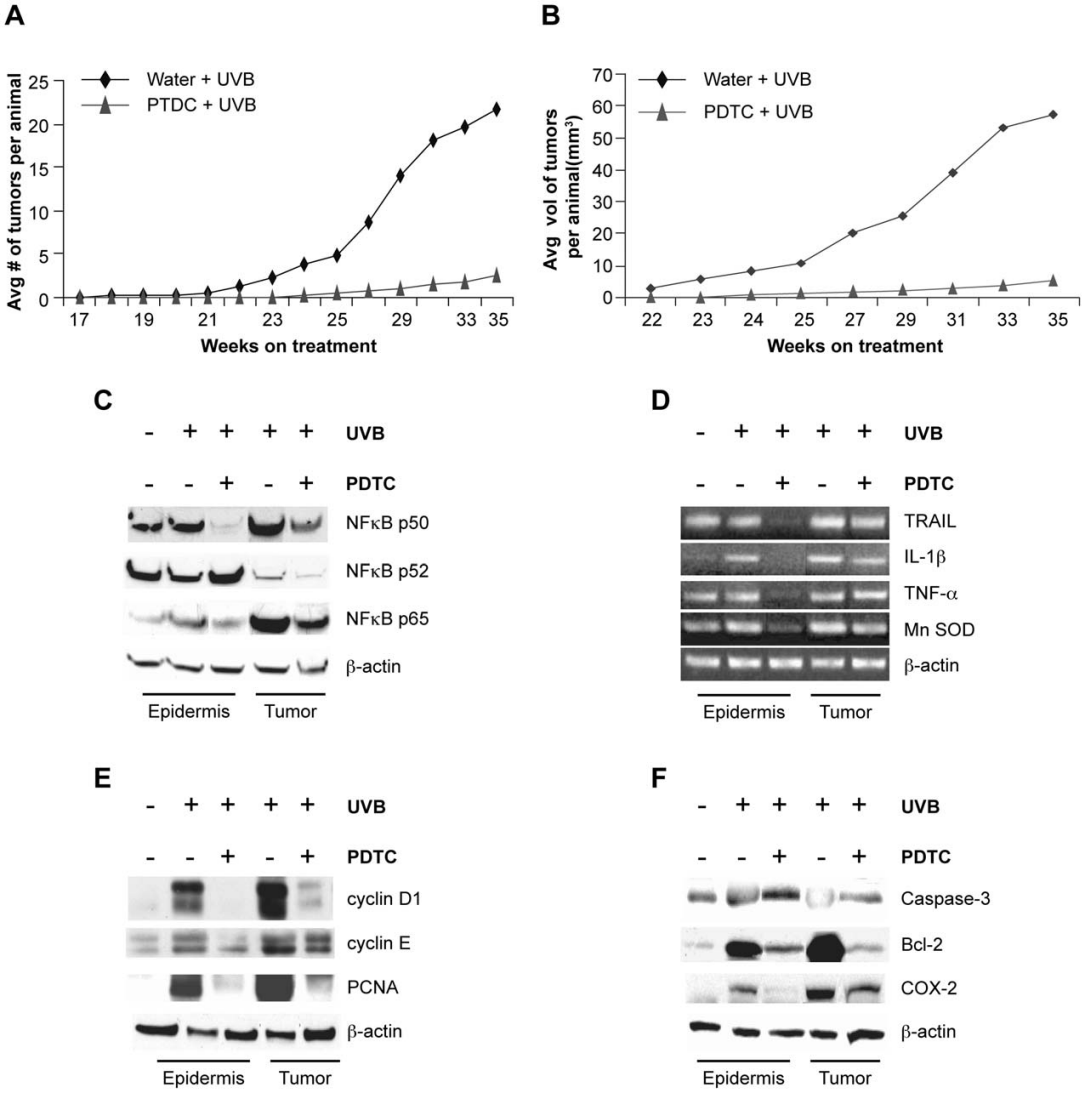
Administration of the NFκB inhibitor PDTC abrogates UVB-induced skin carcinogenesis in SKH-1 mice. SKH-1 mice (n = 30 per treatment) were chronically irradiated with UVB (180 mJ/cm^2^, twice a week for 35 weeks) and either given PDTC (0.5%) or no treatment in drinking water (*ad libitum*). Non-irradiated SKH-1 mice (n = 12) were used as controls. A) PDTC treatment decreased the average number of tumors per mouse. B) PDTC treatment decreased the average tumor volume per mouse. C) Western blot analysis for the p50, p52, and p65 subunits of NFκB were performed on nuclear extracts prepared from non-irradiated epidermal samples and chronically irradiated epidermal and tumor samples from mice treated with PDTC or untreated. While PDTC treatment prevented the upregulation of p50 in both skin and tumor samples, it had no effect on the p52 expression in either sample. PDTC treatment specifically abrogated upregulation of p65 in non-tumor bearing epidermis. D) RT-PCR for the NFκB transcriptional targets TRAIL, IL-1β, TNF-α and Mn SOD was performed on non-irradiated epidermal samples and chronically irradiated epidermis and tumor samples from mice treated with PDTC or untreated. PDTC prevented upregulation of the NFκB targets in irradiated skin samples but not in tumor samples. E) Western blot analysis for cyclin D1, cyclin E, and PCNA (which are involved in cell cycle regulation) was performed on non-irradiated epidermal samples and chronically irradiated epidermis and tumor samples from either untreated mice or mice treated with PDTC. PDTC treatment abrogates the overexpression of these proteins in non-tumor bearing skin samples. F) Western blot analysis of caspase-3 (pro-apoptosis), Bcl-2 (anti-apoptosis), and COX-2 (inflammation) was performed on non-irradiated epidermal samples and chronically irradiated epidermis and tumor samples from mice treated with PDTC or untreated. PDTC inhibited Bcl-2 and COX-2 in both epidermis and tumor samples while it did not affect caspase-3.

We next reasoned that blockade of NFκB signaling *in vivo* should inhibit tumor formation in *Hairless* mutant mice. Therefore, we performed chronic UVB tumor induction studies while simultaneously treating the mice with a pharmacological inhibitor of NFκB, pyrrolidinedithiocarbomate (PDTC), which was given *ad libitum* the mice's drinking water. PDTC suppresses NFκB DNA-binding activity and IκBα degradation thereby inhibiting NFκB activation [34]. 60 SKH-1 mice were divided into two groups: group-I mice received tap water and group-II mice received PDTC (0.5%) supplemented drinking water. All mice were irradiated with UVB, 180 mJ/cm^2^ twice a week for 35 weeks, which was sufficient to induce tumorigenesis both with and without PDTC treatment (Figure 5A and B).

PDTC and its concomitant reduction in NFκB activity significantly reduced tumorigenesis by approximately 60%, as measured in average tumor number, throughout the duration of the irradiation protocol (Figure 5A). Average tumor volume was also significantly decreased in the animals treated with PDTC (Figure 5B). Inhibition of the NFκB pathway altered gross tumor morphology suggesting that the tumors that formed on the PDTC treated animals were more likely to be papillomas than SCCs. In contrast, the animals given only tap water developed signs of aggressive SCCs (Figure S3A). Taken together, these results demonstrate that the tumor susceptibility of the SKH-1 animals is dependent on the overactivation of the NFκB pathway that is a downstream result of *Hairless* mutation.

We next performed a detailed molecular analysis of the NFκB pathway in the PDTC treated SKH-1 animals (Figure 5C-F). For this analysis we used the non-tumor bearing skin and the tumors from the PDTC treated and control SKH-1 animals that had been irradiated for 35 weeks. In addition to analyzing whole tumors in this study, the epidermis was isolated from non-tumor bearing skin in order to localize the biochemical effects of the PDTC treatment. Our initial hypothesis was that non-tumor bearing skin of the PDTC treated animals would exhibit reduced NFκB signaling thereby indicating less tumor promotion when NFκB activation is blocked. Furthermore, we hypothesized that tumors from both untreated and PDTC treated animals would show similar levels of NFκB activity. We reasoned that any tumors formed despite PDTC treatment would suggest the occurance of other molecular events occurred thereby allowing specific cells to bypass PDTC-mediated inhibition and develop into tumors. Therefore, we expected the effects of PDTC and NFκB inhibition in UVB irradiated SKH-1 animals to be more prominent in the non-tumor bearing skin compared to non-treated SKH-1 animals.

### PDTC inhibition of NFκB blocks downstream target genes in chronically UVB exposed *Hairless* mutant skin

We began by assessing NFκB activation by Western blot analysis of nuclear extracts; we found decreased nuclear localization of the NFκB subunits p65 and p50 in non-tumor bearing epidermis and tumors but no change in p52 (Figure 5C). This indicated that as expected, PDTC specifically blocked nuclear translocation of the p65/p50 heterodimer and the canonical NFκB pathway. We also found that PDTC treated epidermis exhibited decreased NFκB activation as measured by direct binding of NFκB to consensus binding sites, electrophoretic mobility shift assay (EMSA)(Figure S3B). Total and phosphorylated IκBα protein levels were measured by Western blot in non-tumor bearing epidermis and tumors; it was found that PDTC treatment specifically abrogated increases in phophorylated IκBα in non-tumor bearing skin suggesting a functional blockade of the NFκB signaling pathway (Figure S3C). Taken together, the data indicates an overall reduction in NFκB signaling activity in the non-tumor bearing skin of SKH-1 animals subjected to chronic UVB exposure and treated with PDTC.

In order to assess the downstream consequences of NFκB regulation by PDTC in SKH-1 animals, we analyzed the mRNA expression of various NFκB target genes in epidermal extracts and tumors using semi-quantitative PCR. As shown in Figure 5D, we found that PDTC treatment significantly reduced the expression of TRAIL [35], IL-1β [36], TNFα [37], and MnSOD [38] in the non-tumor bearing epidermis with a less appreciable decrease in tumors. This data confirms that NFκB activation is crucial for tumor development in chronically UVB irradiated SKH-1 skin, and that PDTC efficiently blocked NFκB activation in non-tumor bearing skin, thus attenuating the susceptibility of SKH-1 animals to tumor development.

To further investigate the PDTC-dependent repression of tumor susceptibility in chronically irradiated *Hairless* mutant mice, we studied its effects on the expression of genes that regulate cell cycle progression, proliferation, and apoptosis. As shown in Figure 5E, PDTC treatment inhibited the expression of the key cell cycle regulators cyclin D1 [39], cyclin E1 [40], and PCNA [41] in both tumors and in non-tumor bearing epidermis. When combined with the increased Ki67 staining seen in Figure 3B, increased expression of these proteins in irradiated SKH-1 skin and tumors is yet another indication of a hyperproliferative state that can be abrogated by blockade of the NFκB pathway.

We then examined the expression of two proteins involved in apoptotic response pathways, the pro-apoptotic caspase 3 and the anti-apoptotic Bcl-2. Caspase 3 has not been implicated in the NFκB pathway so as expected, we saw no significant change in expression with PDTC treatment (Figure 5F). Bcl-2, however, is a known NFκB target gene and its expression is decreased with PDTC treatment in both non-tumor bearing epidermis and tumors (Figure 5F) [42]. This is consistent with the TUNEL and Ki67 results in Figures 3B and 3C that show increased proliferation and decreased apoptosis, which together with the data presented here, illustrates that repression of NFκB restores the normal apoptotic response in the irradiated *Hr* mutant epidermis.

To further understand the mechanism behind increased tumor promotion in the context of NFκB activation, we assessed the expression of the pro-inflammatory protein COX-2. COX-2 is known to be downstream of NFκB and is strongly implicated in squamous cell carcinoma progression [43], [44], [45]. In agreement with the expression pattern of the other pro-inflammatory NFκB target genes TNFα and IL-1β, we found that COX-2 levels were dramatically decreased with PDTC treatment in non-tumor bearing skin but not in tumors. Therefore, we theorize that in the *Hr* mutant epidermis, increased COX-2 levels in conjunction with TNFα and IL-1β create an pro-inflammatory environment that promotes tumor growth [46].

## Discussion

Hairless mice, particularly the SKH-1 line, have long been a favorite animal model used for skin carcinogenesis studies because of their convenience, however, researchers have not generally taken into consideration the underlying mechanism for their lack of hair and whether this may be linked to their tumor susceptibility. To address this issue, we show for the first time that *Hr^−/−^* and SKH-1 mouse lines are allelic mutants of one another that have significantly lower amounts of *Hr*, a crucial early UVB response gene, and are thus predisposed to UVB induced carcinogenesis. The results detailed above indicate that Hr plays a crucial role in cell cycle kinetics by functioning as an inhibitor of the NFκB pathway in the epidermis; therefore, loss of *Hr* results in altered cell cycling, increased proliferation and subsequent tumorigenesis.

We found that both *Hr^−/−^* and SKH-1 animals exhibit similar decreases of *Hr* expression throughout the skin that amounts to approximately 30% of that found in WT animals; this allowed them to be used interchangeably in our experiments. We further determined that *Hr* is significantly upregulated in the WT epidermis 8 hours after a single, acute dose of UVB. Furthermore, when *Hr^−/−^* mice were used in chronic UVB carcinogenesis studies they developed papillomas by week 30, and these papillomas progressed into aggressive SCCs by the end of the protocol 20 weeks later. In contrast, WT mice with normal *Hr* expression failed to show even microscopic signs of tumorigenesis by week 50.

Together, these findings led us to postulate that Hr is an essential component of the epidermal response to UVB insult and perturbation of *Hr* levels causes a genetic predisposition to tumorigenesis due to a dysregulated UVB response in the *Hr* mutant epidermis. Under normal conditions, a tightly regulated signaling cascade is triggered by UVB irradiation leading to the apoptosis of severely damaged cells and proliferation of undamaged and/or repaired cells (reviewed in [47]). Therefore, we examined the epidermis of *Hr^−/−^* animals and found that at homeostasis *Hr* loss results in a significantly thicker epidermis that is sustained after UVB exposure. We then investigated the contribution of both proliferation and apoptosis to this thicker epidermis and found both increased proliferation at all time points and decreased apoptosis in response to UVB irradiation. This indicated that the thicker epidermis found in *Hr* mutant mice was the result of uncontrolled proliferation and decreased apoptosis, factors which when combined together contribute to the increased tumorigenesis seen during the chronic UVB exposure protocol. It should be noted that the thicker epidermis in and of itself might contribute to the decreased apoptosis due to possible UVB shielding.

One of the key mechanistic links between proliferation and apoptosis is the NFκB signaling pathway and we have previously established a regulatory loop between Hr and NFκB *in vitro* [29], [46]. Thus we investigated a possible mechanistic connection between NFκB and Hr *in vivo*. While there are many NFκB hetero- and homo-dimers that can activate NFκB-mediated transcription and regulate cell proliferation, we began by focusing on the canonical p65/p50 hetero-dimer complex since it is known to be an important UVB response factor in both mouse and human skin [24]. We found that at homeostasis, *Hairless* mutant epidermis exhibits constitutive activation of the NFκB pathway as indicated by increased expression of both the p65 subunit and p-IκBα. Unlike in the WT epidermis, p-IκBα activation in the *Hr^−/−^* skin is not confined to the superbasal layer; instead p-IκBα is expressed throughout the epidermis both basely and in response to UVB thererby indicating aberrant upregulation of NFκB in *Hr^−/−^* skin.

We postulate that Hr regulates tumor promotion via regulation of NFκB activation and thereby modulates proliferation and apoptosis following UVB irradiation. Therefore, when Hr expression is decreased, there is an increased likelihood of the survival and proliferation of UVB damaged basal keratinocytes and subsequent tumorigenesis. As we also show here, blockade of the NFκB pathway by the pharmacological inhibitor PDTC decreases both tumor burden and tumor volume in the *Hairless* mutant mouse line SKH-1.

In the non-tumor bearing skin irradiated SKH-1 mice, PDTC treatment specifically decreased NFκB activity in the *Hairless* mutant epidermis as reflected by attenuated expression of the NFκB subunits p50 and p65, decreased NFκB binding to target sequences, and decreased expression of p-IκBα. Additionally, the non-tumor bearing skin of PDTC treated mice also exhibited decreases in the expression of downstream NFκB targets such as TRAIL, IL-1β, TNFα, and MnSOD as well as the inhibition of cell cycling proteins such as cyclin D1, cyclin E, PCNA, and BCL-2 thereby indicating a return to normal proliferation. We also show that in non-tumor bearing skin, *Hr* influences inflammation via COX-2 expression which decreases with PDTC treatment. The combination of decreased TNFα, IL-1β, and COX-2 found in the PDTC treated epidermis provides compelling evidence that *Hr* is capable of modulating epithelial inflammation via regulation of the NFκB pathway and thereby further contributes to tumor promotion. Therefore, the data provides evidence that the decreases in *Hr* expression result in gross epithelial dysregulation that can be ameliorated by inhibition of the NFκB pathway.

We hypothesize that since NFκB is the nexus of proliferative and inflammatory signals [46], the lack of *Hr* allows the proliferation of UVB damaged cells and eventual tumor development. Previous work in our laboratory has shown that after UVB insult, cells lacking *Hr* have decreased amounts of cells in the G2/M phase [29] which is a key cell cycle checkpoint for DNA damage [48], [49]. Attenuation of the G2/M checkpoint after UVB insult increases genomic instability which when combined with increased proliferation and inflammation found in the *Hairless* mutant epidermis creates an ideal cellular environment for tumor development. Thus, loss of *Hr* and subsequent overactivation of NFκB provide all the components necessary for increased tumorigenesis.

While it is possible that Hr-mediated repression of the NFκB pathway could occur at multiple junctures due to the inherent complexities of the NFκB pathway, the evidence presented here suggests the involvement of Hr in two possible regulatory mechanisms. One is through the control of the kinase cascade leading to IκBα phosphorylation and subsequent degradation. This then allows the canonical NFκB heterodimer to translocate to the nucleus, bind to target DNA sequences, and modulate downstream gene transcription. This possibility is seen in the increased IκBα staining seen in *Hr^−/−^* epidermis both prior to and after irradiation. The other possible regulatory mechanism is through direct transcriptional regulation of the p65 subunit. Evidence for this regulation is seen in the increased expression of p65 in epidermal extracts from unirradiated and irradiated *Hr^−/−^* mice. Since IκBα is also downstream of NFκB activation, direct transcriptional regulation of the NFκB subunits would also result in the increased IκBα phosphorylation seen in the *Hr* mutant epidermis. Therefore either hypothesis fits the data presented here and further work will be necessary to clarify the method of Hr regulation of NFκB.

While non-tumor bearing skin showed significant changes with PDTC, there were less obvious effects in the tumor samples. We postulate that specific keratinocyte populations escaped PDTC-mediated inhibition of NFκB and developed into tumors despite PDTC treatment. We further postulate that this escape may be due to other molecular events such as DNA damage in other genes downstream of PDTC inhibition of the NFκB pathway. Therefore, the effects of PDTC are more prominent in the non-tumor bearing skin and a small number of tumors develop despite PDTC treatment.

Altogether, we provide here multiple lines of evidence that Hr plays an essential role in the regulation of NFκB signaling in the epidermis and thereby effects keratinocyte apoptosis and proliferation. Hr mediated NFκB regulation occurs during epidermal homeostasis but it is particularly important during the keratinocyte response to UVB irradiation where it is essential to the proper apoptosis of damaged keratinocytes. Thus, we postulate that Hr plays a crucial regulatory role in SCC development via inhibition of NFκB pathway after UVB irradiation. We conclude that *Hr* mutant mouse strains, including SKH-1, exhibit qualities associated with increased proliferation and cellular dysregulation, providing a molecular mechanism for their inherent tumor susceptibility.

## Materials and Methods

### Animals

SKH-1 mice were purchased from Charles River Laboratories (Kingston, NY). HRS/J (*Hr^−/−^*) were purchased from Jackson Laboratory (Bar Harbor, ME). This study was carried out in strict accordance with the recommendations in the Guide for the Care and Use of Laboratory Animals of the National Institutes of Health. The protocol was approved by the Institutional Animal Care and Use Committee of Columbia University (IACUC Protocol Number: AC-AAAA5875).

### RNA and Protein Extraction

Dorsal skin was harvested from mutant and WT animals and divided into two parts; one portion was kept intact for histological analysis and RNA extraction (see below) and the other was used for epidermal isolation and subsequent RNA and protein extraction. Briefly, dorsal whole skin was scraped to remove excess subcutaneous fat and divided into small strips and incubated in 0.25% trypsin with EDTA (Invitrogen, Grand Island, NY) for 2 hrs at 37°C. The epidermis was mechanically separated from the dermis by scraping and incubated in SMEM with 10% FBS, 1% PS (Invitrogen) at room temperature with shaking for 30 min to separate the keractinoyctes. Keratinocyte clumps were then filtered out using a 70 μM cell strainer (BD Biosciences, San Diego, CA) and the remaining single cell filtrate was pelleted by centrifugation. The cells were then resuspended in either RLT Buffer for RNA Extraction (Qiagen, Valencia, CA) or the Flag-Lysis Buffer (50 mM Tris-HCl pH 7.8, 137 mM NaCl, 10 mM NaF, 1 mM EDTA, 1% Triton X-100, 0.2% Sarkosyl, 1 mM DTT, 10 % glycerol and fresh proteinase inhibitors) for protein extraction.

A portion of the whole skin was also homogenized in RLT Buffer and RNA was isolated from either the epidermis or the whole skin using an RNeasy Mini Kit (Qiagen) followed by DNase treatment. 2 μg of total RNA was used for the cDNA synthesis using Superscript III (Invitrogen) and a 2∶1 ratio of random hexamers: oligo (dT) primers according to manufacturer's instructions.

Protein was extracted from either epidermal extracts or whole tumors homogenized in Flag-Lysis buffer. Proteins were quantified using RD DC protein assay protocol as instructed by the manufacturer (BioRad, Hercules, CA). Relative band intensity was measured using ImageJ software.

### Quantitative RT-PCR

Real time PCR was performed on a ABI 7300 (Applied Biosystems, Carlsbad, CA) using the Relative Quantification Assay and software. Primers (Invitrogen) were designed according to ABI guidelines.

All are written 5′ to 3′:

m*Hr* ex 6 FTTCCACATTGCTGCAGTCAT

m*Hr* ex 6/7 RGTTCTCAGG AACAGCA

m*Hr* ex15 FCTGGTATCGAGCACAGAAAG

m*Hr* ex 16 FCTTTCTCCAGATGGTGTGC

m*B2M* FACTGACCGGCCTGTATGCTA

m*B2M* RTAGAGATGTCAGATATGTCCTTCA

mTNF-α FTGCCTATGTCTCAGCCTCTT

mTNF-α R****GGAAGACTCCTCCCAGGTAT

mIL-1β FATAACCTGCTGGTGTGTGAC

mIL-1β RTGAGGTGCTGATGTACCAGTT

mMnSOD FGACCTGCCTTACGACTATGG

mMnSOD R GACCTTGCTCCTTATTGAAGC

mTRAIL FTCACCAACGAGATGAAGCAGC

mTRAIL RCTCACCTTGTCCTTTGAGACC

All reactions were performed using ABI Power Sybr Master Mix, 200nM primers and 200ng cDNA. The following protocol was used: step 1 −50°C 2 min, step 2 −95°C 10 min, step 3 −95°C 15 sec, step 4 −60°C 1 min, repeat step 2–4 for 40 cycles. All samples were run on a 1.5% agarose gel to confirm amplicon size. The data output was imported into the GraphPad Prism 4 software (GraphPad Software, Inc., La Jolla, CA), plotted, and the error calculated as standard error of the mean (SEM). Statistical analysis was performed between time points and samples as indicated using Student's T-test.

### UV irradiation protocol

For all protocols, irradiation was performed using eight FS72T12-UVB-HO lamps (Daavlin, Bryan, OH) and all animals were irradiated at 180 mJ/cm^2^ UVB which has been established as approximately equivalent to 2 MEDs [30], [31]; UVC was eliminated by a Kodacel filter (TA401/407) (Kodak, Rochester, NY). The UVB dose was measured with a UVB Spectra 305 Dosimeter (Daavlin) and calibrated with a secondary radiometer (IL1700) (International Light, Peabody, MA).

#### Chronic UVB protocol

Female 9–11 week old HRS/J and shaved WT counterparts (n = 10 for each) were irradiated three times a week for a total of 50 weeks. Tumors were counted when they reached 2 mm in diameter on a weekly basis. After 25 and 30 weeks of irradiation mice were randomly selected for euthanization. At 42 weeks of irradiation mice were chosen for sacrifice based on their tumor size; any animal with an average tumor diameter greater than 7.5 mm was euthanized. After 50 weeks of irradiation the remaining mice were sacrificed. At all timepoints skin and tumor samples were embedded in both paraffin and frozen blocks.

#### Chronic UVB protocol with PDTC treatment

60 9–11 week old SKH-1 mice were split into two groups of 30 animals each, one group received no treatment and the other group received 0.5% PDTC in their drinking water. PDTC treatment began 2 weeks prior to first irradiation and continued throughout irradiation protocol. Both groups were irradiated twice a week with for a total of 35 weeks. 12 SKH-1 mice served as non-irradiated, untreated, age matched controls. When tumor diameter reached 2 mm, the tumor number and size were recorded biweekly. All animals were sacrificed 24 hrs after last UVB exposure and dorsal skin and tumors were collected for Western blotting, EMSA and RT-PCR analysis. Three samples for each group was used per assay and each assay was repeated for a total of three times.

**Acute UVB protocol.** Female 9–11 week old HRS/J and shaved WT counterparts were irradiated once. Three animals of each group per post irradiation timepoint were used for 4 replicates (n = 12).

### Immunohistochemistry

Skin samples for frozen blocks were prepared and embedded as previously described [50]. Sections were done at 4 μm, parallel to the paravertebral line, fixed in buffered 4% paraformaldehyde with 1% Triton-X and processed for H&E, TUNEL (Apo-Tag kit) (Chemicon, Billerica, MA) and Ki-67 (AbCam, Cambridge, MA) staining. Epidermal morphometry was measured on H&E stained sections using AdobePhotoshop. Approximately 20 measurements (from basement membrane to stratum corneum) were made per 2.5 mm section at 10X objective on a HRC Axiocam fitted onto an Axioplan2 fluorescent microscope (Carl Zeiss, Thornwood, NY). TUNEL and Ki67 positive cells were counted per 2.5 mm section of skin at 20X. For Ki67 analysis the total number of basal keratinocytes were also counted in the same field of view. Data was plotted and analyzed using GraphPad Prism 4 software. Error was calculated as standard error of the mean (SEM).

For phospho-IκBα staining, tissue was fixed in 10% formalin, embedded in paraffin blocks and sectioned at 4 μm parallel to paravetebral line following standard procedures. Sections were treated with Antigen Unmasking Solution (Vector Labs, Burlingame, CA) then probed with phospho-IκBα (ser32/36) (AbCam) followed by avidin-biotintylated HRP complex (ABC Elite system, Vector Labs). The antibody was detected with DAB and images were taken using a HRC Axiocam fitted onto an Axioplan2 fluorescent microscope.

### Western Blots

Protein extracts were prepared as described above, while nuclear extracts were prepared using NE-PER Nuclear and Cytoplasmic Extractions kit (Pierce, Rockford, IL). All samples were used for Western blotting analysis using standard protocols and the following primary antibodies: p65 (AbCam), p50 (Santa Cruz, Santa Cruz, CA), and p52 (Santa Cruz). Western blotting was done using standard protocols and the following primary antibodies: IκBα (Santa Cruz) IκBα (ser32/36) (AbCam), PCNA (Santa Cruz), Bcl-2 (Enzo Life Sciences, Farmingdale, NY), active caspase 3 (AbCam), Cyclin D1 (Cell Signaling Technology, Boston, MA), Cyclin E (Santa Cruz), COX-2 (Santa Cruz), and β-actin (Sigma-Aldrich, St Louis, MO). Appropriate horseradish-peroxidase conjugated secondary antibodies (Jackson ImmunoResearch, West Grove, PA) were used at 1∶1000 and visualized using SuperSignal West Dura Chemiluminescent Substrate (Pierce, Rockford, IL). Relative band intensity was measured using ImageJ (NIH, Bathesda, MD).

### EMSA

3.5 pmol of NFκB consensus oligonucleotide (5′- AGTTGAGGGGACTTTCCCAGGC-3′) (Promega, San Louis Obispo, CA) was 5' labeled with [γ-32P] ATP using T4 polynucleotide kinase (Promega). Nuclear protein extracts were incubated in Gel Shift Binding Buffer (Promega) containing 20% glycerol (v/v), 5mM MgCl_2_, 2.5mM EDTA, 2.5mM DTT, 250mM NaCl and 50mM Tris-HCl (pH 7.5) and labeled oligonucleotide. In competition experiments, a 100-fold molar excess of unlabeled probe was added before the labeled probe. Reaction products were separated on 4% a polyacrylamide gel, after which the gel was dried and exposed to X-ray film overnight at −70°C.

## Supporting Information

Figure S1 **SKH-1 and** ***Hr^−/−^*** **mice contain the same proviral insertion**. A) Schematic layout of primer locations and insertion site. B) Using PCR primers designed to intron 5F and intron 6R1 (lane1) yields a product in all genotypes, while primers designed to intron6F, mid intron6R2 (lane2), and exon6/intron6F, intron6R3 (lane3) amplifies only in the WT samples indicating an insertion is present causing the PCR to fail. C) Using *Hairless* specific Hairless primer (ex6/intron6F) and viral specific reverse primers (lane3), products were observed only in the mutants indicating SKH-1 and *Hr^−/−^* have the same viral insertion. D) DNA sequencing was performed to confirm the insertion as the same as that found in *Hr^−/−^*. Black arrow indicates the insertion site junctions.
(TIF)

Figure S2 ***Hr^−/−^*** **animals are unaffected by chronic UVB irradiation until 30 weeks**. A) At 22 weeks of chronic UVB irradiation, neither WT nor *Hr^−/−^* animals show any signs of tumorigenesis. B) By 30 weeks of chronic UVB irradiation, *Hr^−/−^* animals begin to develop small papillomas.
(TIF)

Figure S3 **PDTC treatment prevents tumorigenesis and activation of NFκB in SKH-1 animals**. A) After 35 weeks of chronic UVB irradiation, SKH-1 animals given only tap water develop tumors with the gross morphology of aggressive SSCs. PDTC treated SKH-1 animals at the same time point develop significantly less tumors that appear to be less aggressive. B) EMSA using samples derived from unirradiated epidermis, non-tumor bearing epidermis from untreated and PDTC-treated animals, and tumors from untreated and PDTC-treated animals. PDTC treatment significantly decreases NFκB binding to target sequences (indicated by black arrow). C) Western blot of IκBα and p-IκBα from unirradiated epidermis, non-tumor bearing epidermis from untreated and PDTC-treated animals, and tumors from untreated and PDTC-treated animals. PDTC treatment decreased p-IκBα expression in the non-tumor bearing skin of irradiated animals.
(TIF)
