# Supplementary figures and images for: Loss of Hairless Confers Susceptibility to UVB-Induced Tumorigenesis via Disruption of NF-kappaB Signaling
Source: PLoS One. 2012 Jun 25;7(6):e39691. doi: 10.1371/journal.pone.0039691 (PMC3382590; doi:10.1371/journal.pone.0039691)

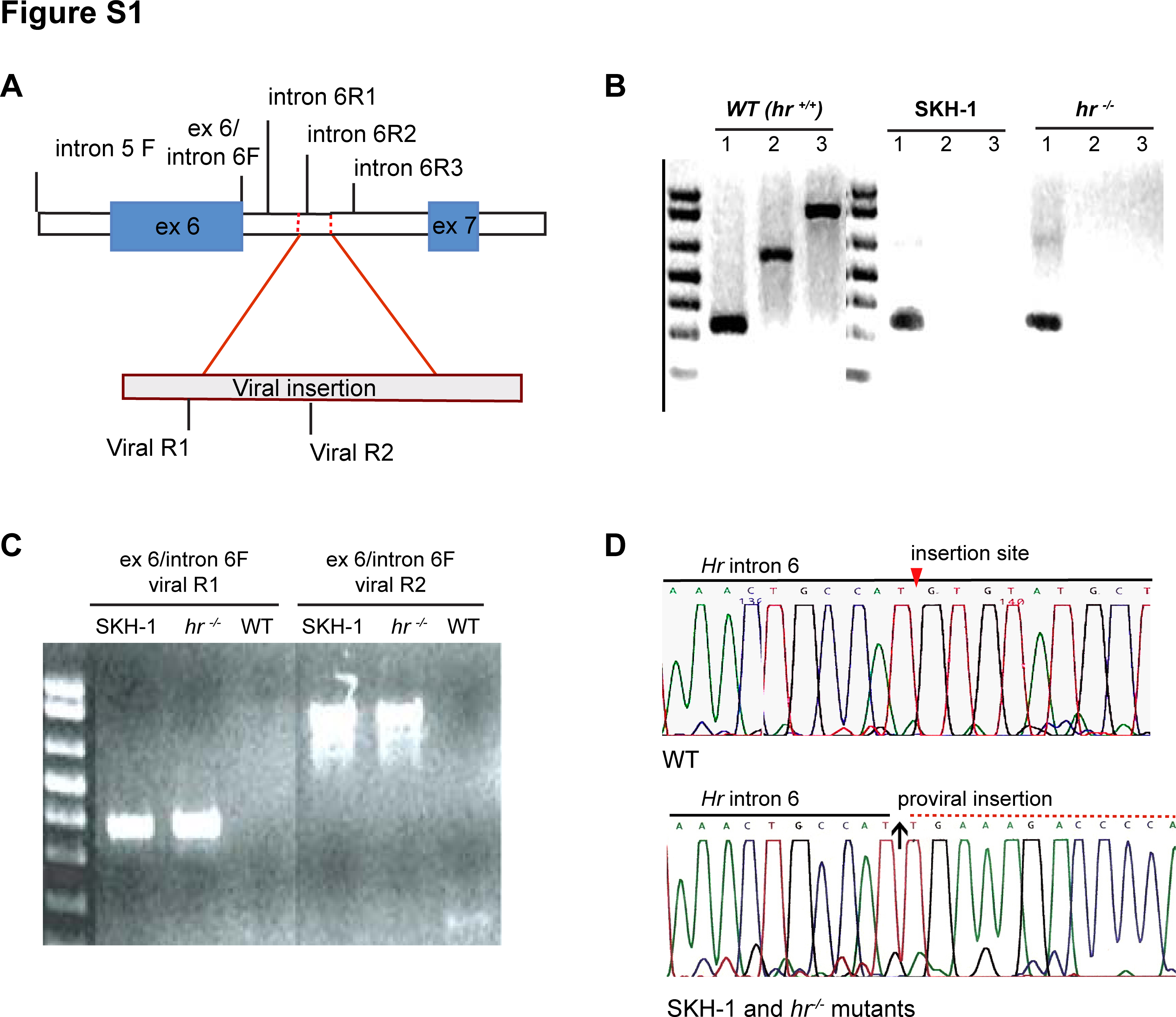

Supplement: Figure S1 — SKH-1 and Hr−/− mice contain the same proviral insertion. A) Schematic layout of primer locations and insertion site. B) Using PCR primers designed to intron 5F and intron 6R1 (lane1) yields a product in all genotypes, while primers designed to intron6F, mid intron6R2 (lane2), and exon6/intron6F, intron6R3 (lane3) amplifies only in the WT samples indicating an insertion is present causing the PCR to fail. C) Using Hairless specific Hairless primer (ex6/intron6F) and viral specific reverse primers (lane3), products were observed only in the mutants indicating SKH-1 and Hr−/− have the same viral insertion. D) DNA sequencing was performed to confirm the insertion as the same as that found in Hr−/−. Black arrow indicates the insertion site junctions. (TIF) [file pone.0039691.s001.tif]

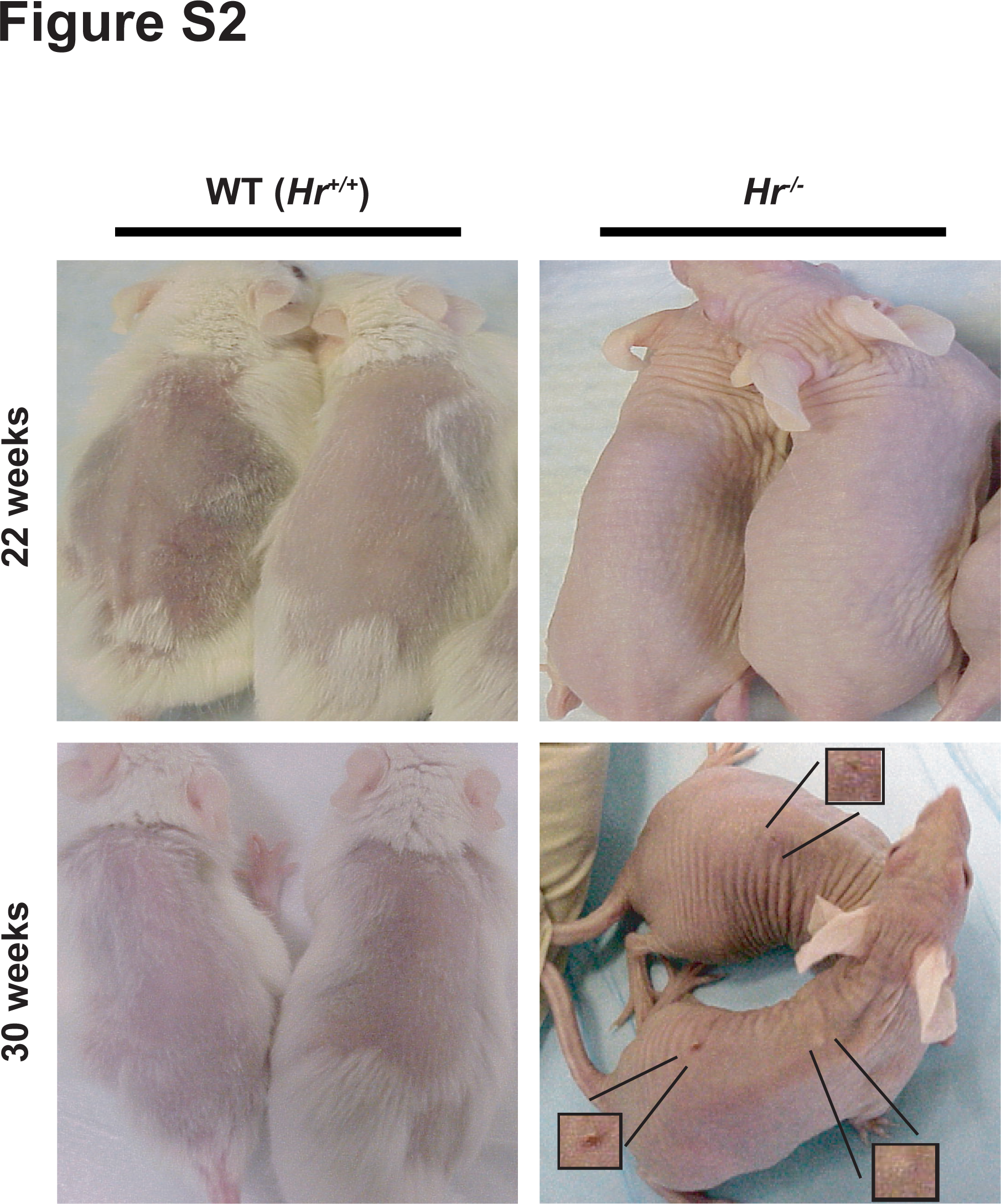

Supplement: Figure S2 — Hr−/− animals are unaffected by chronic UVB irradiation until 30 weeks. A) At 22 weeks of chronic UVB irradiation, neither WT nor Hr−/− animals show any signs of tumorigenesis. B) By 30 weeks of chronic UVB irradiation, Hr−/− animals begin to develop small papillomas. (TIF) [file pone.0039691.s002.tif]

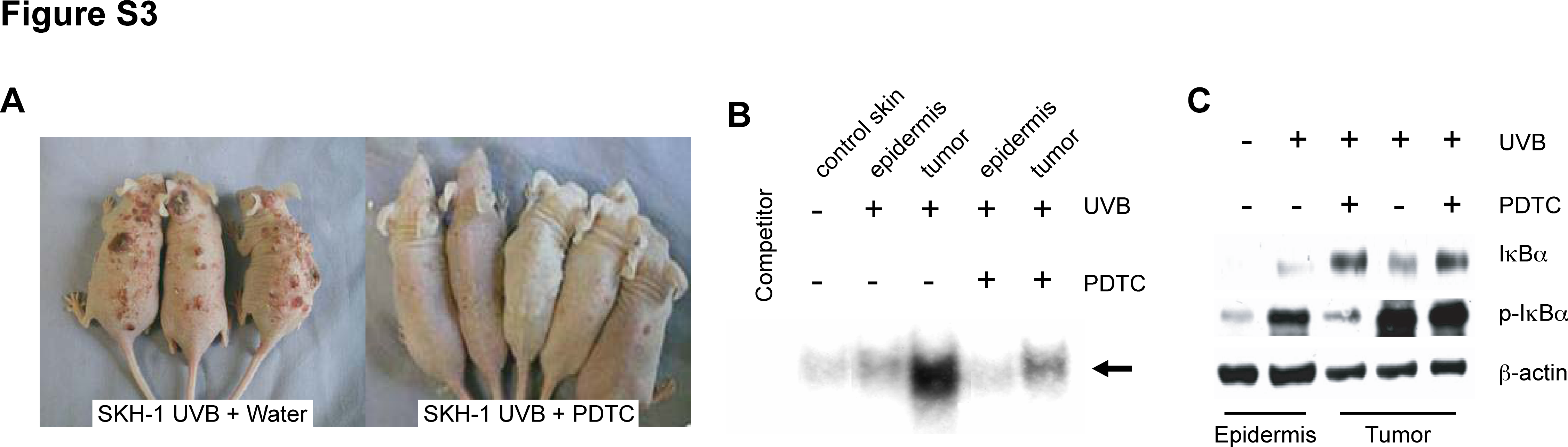

Supplement: Figure S3 — PDTC treatment prevents tumorigenesis and activation of NFκB in SKH-1 animals. A) After 35 weeks of chronic UVB irradiation, SKH-1 animals given only tap water develop tumors with the gross morphology of aggressive SSCs. PDTC treated SKH-1 animals at the same time point develop significantly less tumors that appear to be less aggressive. B) EMSA using samples derived from unirradiated epidermis, non-tumor bearing epidermis from untreated and PDTC-treated animals, and tumors from untreated and PDTC-treated animals. PDTC treatment significantly decreases NFκB binding to target sequences (indicated by black arrow). C) Western blot of IκBα and p-IκBα from unirradiated epidermis, non-tumor bearing epidermis from untreated and PDTC-treated animals, and tumors from untreated and PDTC-treated animals. PDTC treatment decreased p-IκBα expression in the non-tumor bearing skin of irradiated animals. (TIF) [file pone.0039691.s003.tif]
